# Single-Chain Magnets Based on Octacyanotungstate with the Highest Energy Barriers for Cyanide Compounds

**DOI:** 10.1038/srep24372

**Published:** 2016-04-13

**Authors:** Rong-Min Wei, Fan Cao, Jing Li, Li Yang, Yuan Han, Xiu-Ling Zhang, Zaichao Zhang, Xin-Yi Wang, You Song

**Affiliations:** 1 State Key Laboratory of Coordinate Chemistry, Collaborative Innovation Center of Advanced Microstructures, School of Chemistry and Chemical Engineering, Nanjing University, Nanjing 210093, China; 2 Key Laboratory of Coordination Chemistry and Functional Materials in Universities of Shandong, School of Chemistry and Chemical Engineering, Dezhou University, Dezhou 253023, China; 3 Jiangsu Key Laboratory for the Chemistry of Low-Dimensional Materials, School of Chemistry and Chemical Engineering, Huaiyin Normal University, Huai’an 223300, China

## Abstract

By introducing large counter cations as the spacer, two isolated 3, 3-ladder compounds, (Ph_4_P)[Co^II^(3-Mepy)_2.7_(H_2_O)_0.3_W^V^(CN)_8_]·0.6H_2_O (**1**) and (Ph_4_As)[Co^II^(3-Mepy)_3_W^V^(CN)_8_] (**2**, 3-Mepy = 3-methylpyridine), were synthesized and characterized. Static and dynamic magnetic characterizations reveal that compounds **1** and **2** both behave as the single-chain magnets (SCMs) with very high energy barriers: 252(9) K for **1** and 224(7) K for **2**, respectively. These two compounds display the highest relaxation barriers for cyano-bridged SCMs and are preceded only by two cobalt(II)-radical compounds among all SCMs. Meanwhile, a large coercive field of 26.2 kOe (**1**) and 22.6 kOe (**2**) were observed at 1.8 K.

Since the first observation of the Glauber dynamics^1^ in a one-dimensional coordination polymer^2^, the single-chain magnets (SCMs) named in 2002^3^ have attracted considerable attention owing to their potential applications in quantum computing, spintronics, and high-density memory devices^3^,^4^,^5^. For the rational design of SCMs of high relaxation barrier and high blocking temperature (*T*_B_), strong intrachain magnetic coupling and spin carriers of large magnetic anisotropy are of crucial importance. A variety of SCMs have been synthesized from specific anisotropic metal ion and bridging ligands^6^,^7^,^8^,^9^,^10^,^11^,^12^,^13^,^14^. Among all the bridging ligands, the CN group is a very efficient bridge mediating strong magnetic interaction between metal ions and has played a prominent role in the development of SCMs research^5^,^15^,^16^,^17^,^18^,^19^,^20^. Following the first cyano-bridged SCM^21^ reported in 2003, more and more cyano-bridged SCMs have been synthesized and characterize^15^,^16^,^17^,^18^,^19^,^20^,^21^,^22^,^23^,^24^,^25^.

Octacyanometallates [M^IV/V^(CN)_8_]^4−/3−^ (M = Nb, Mo, W) are of great importance in the molecule-based magnets^26^,^27^,^28^,^29^,^30^. The stereochemical flexibility of these building blocks facilitates the construction of various topologies and magnetic properties^26^,^27^,^28^,^29^,^30^. Compared with the widely studied hexacyanometallates, octacyanometallates possess a slightly weaker coordination ability to the first row transition metal ions, which facilitates the construction of low dimensional magnetic materials. In addition, the more radially extended valence orbitals of the 4d/5d metal centers might efficiently strengthen the exchange interactions^31^. However, octacyanometallate-based SCMs are scarcely reported^17^,^32^,^33^, probably due to the difficulty to effectively control the valence of [M^IV/V^(CN)_8_]^4−/3−^ for the design and synthesis of 3d-4d/5d heteronuclear 1D chains.

Although the SCM-based magnets showing both the long range magnetic ordering and SCM behavior have added new aspects to the SCM researches^34^,^35^, a pure SCM with negligible interchain magnetic interaction is still preferred. Synthetically, in order to isolate the 1D Ising magnetic chains, several strategies have been utilized. Among them, large counter ions are considered preferentially to effectively separate the chains and minimize the interchain interaction. For example, in the famous [Mn_2_Ni] SCM family reported by Clérac and Miyasaka *et al.*, the 1D [Mn_2_Ni] chains were isolated by various anions such as ClO_4_^−^, PF_6_^−^, BF_4_^−^, ReO_4_^−^, and BPh_4_^−^, leading to the modification of their magnetic properties^3^,^34^,^36^,^37^. In 2005, we reported the first SMMs based on the 4d/5d metal centers, namely Co^II^[Co^II^(CH_3_OH)_3_]_8_[M^V^(CN)_8_]_6_· Solv (M = Mo, W)^38^. These SMMs suggested the feasibility of using the Co^II^ ion and the octacyanometallates for the construction of low dimensional magnetic materials of strong magnetic anisotropy. Inspired by aforementioned considerations, we devoted to the preparation of the SCMs using the [M^V^(CN)_8_]^3−^ units and sterically bulky cations, such as the Ph_4_P^+^ and Ph_4_As^+^ cations. Here, we report the successful construction of two cyano-bridged 1D ladder chain compounds, or so-called 3, 3-ladder chain^39^: (Ph_4_P)[Co^II^(3-Mepy)_2.7_(H_2_O)_0.3_W^V^(CN)_8_]·0.6H_2_O (**1**) and (Ph_4_As)[Co^II^(3-Mepy)_3_W^V^(CN)_8_] (**2**). As expected, compounds **1** and **2** exhibit SCM behavior with high energy barriers. Most interestingly, both their energy barriers and the blocking temperatures are the highest for all the cyano-bridged SCMs. In addition, the barriers of **1** and **2** are only next to the cobalt(II)-radical compounds^40^,^41^ reported by Maria G. F. Vaz *et al.* among all SCMs.

## Results

### Crystal Structures Description

**Figure 1:**
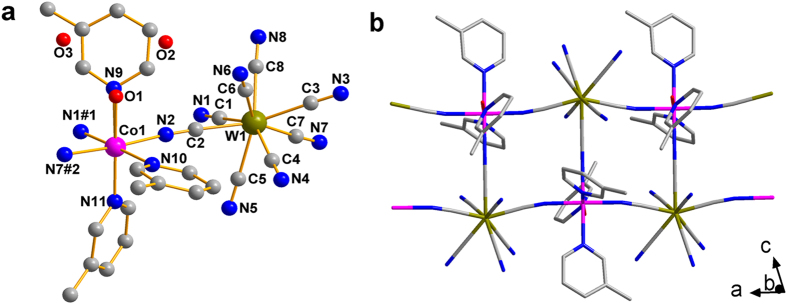
Molecular structure of crystal 1. (**a**) The asymmetric unit of compound **1**. (**b**) The 1D 3, 3-ladder along the *a* axis. Hydrogen atoms and Ph_4_P^+^ cations were omitted for clarity. Symmetry code: #1 = 2*−x*, *−y*, 1*−z*; #2 = *x*−1, *y*, *z.*

Single crystal X-ray diffraction analysis revealed that **1** and **2** are isomorphous and crystallized in the triclinic 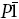 space group (Supplementary Table S1). Both compounds are made up of anionic cyano-bridged Co-W 3, 3-ladder chains running along the *a* axis (Fig. 1), Ph_4_P^+^ (for **1**) or Ph_4_As^+^ (for **2**) counter cations and lattice water molecules (for **1**). Although **1** and **2** are isomorphous, disorder was only found in **1**. One of the three 3-Mepy ligands of **1** has a site occupancy fact of only 0.7, while the rest 0.3 is occupied by a water molecule. Thus, each Co^II^ center is in a distorted octahedral N_6_ environment with six nitrogen atoms from three [W^V^(CN)_8_]^3−^ units and three 3-Mepy (for **1** and **2**) or in a N_5_O environment from two 3-Mepy, one bound water molecule and three [W^V^(CN)_8_]^3−^ ions (for **1**). All three coordinated CN groups from [W^V^(CN)_8_]^3−^ are in the meridional position of the octahedron. The Co–N or Co–O bond lengths and the N–Co–N or N–Co–O bond angles are all in the normal range for a slightly distorted octahedron (Supplementary Tables S2 and S3). The continuous shape measures (CShMs) relative to the ideal octahedron calculated using the program SHAPE 2.1^42^ was 0.253 (CoN_6_)/0.282 (CoN_5_O) and 0.236 for the Co^II^ centers of **1** and **2**, respectively. On the other hand, the polyhedral shape around the W centers does not correspond to ideal symmetry and the [W^V^(CN)_8_]^3−^ unit shows a distorted dodecahedral configuration (TDD) for both **1** and **2** with the CShMs to be 0.56 and 0.66 (Supplementary Table S4). The W–C and C–N bond lengths and the W–C–N bond angles are also consistent with the reported values (Supplementary Tables S2 and S3).

Each [W^V^(CN)_8_]^3−^ unit uses only three cyanides groups in a relatively T-shaped position to connect to three neighboring Co^II^ centers, with the intrachain Co–W distances being 5.32, 5.38, 5.38 Å for **1** and 5.34, 5.39, 5.41 Å for **2**, respectively. Bridged by these cyanide groups, a one-dimensional Co–W chain is formed along the *a* axis. Within the chain, both the Co^II^ and W^V^ centers connect to three neighbors and this kind of chain can be described as a 3, 3-ladder with regularly alternating W^V^ and Co^II^ along the edges^39^. However, as both the Co–N–C and W–C–N bond angles and the torsion angles of the W–C–N–Co connection deviate from 180° (Supplementary Tables S2 and S3), the 3, 3-ladders for both **1** and **2** are slightly distorted. Separated by the bulky Ph_4_P^+^ or Ph_4_As^+^ cations and 3-Mepy ligands (Supplementary Fig. S2), these chains are well isolated to each other, with the shortest interchain Co···Co, Co···W, and W···W distances being 12.8, 11.1, 12.8 Å for **1** and 13.0, 12.9, 13.0 Å for **2**, respectively. These large distances will efficiently prevent any interchain magnetic coupling and lead to the SCM behavior of these compounds, as described below.

### Magnetic Properties

**Figure 2:**
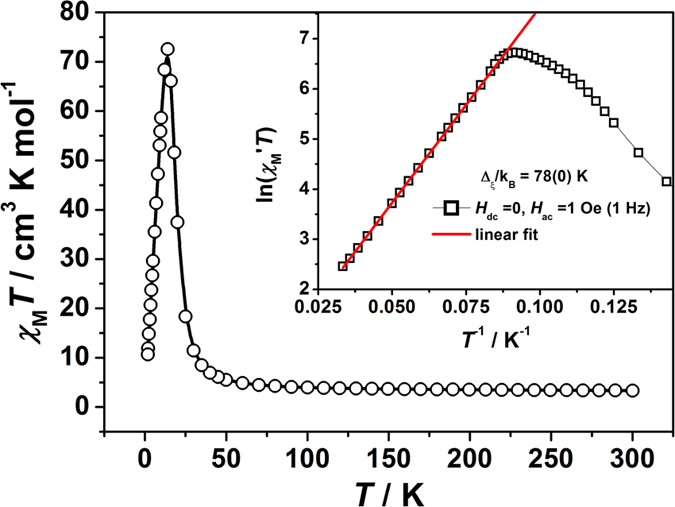
Variable-temperature dc magnetic susceptibility data in the form of *χ*_M_*T* for 1. Measured in an applied field of 2 kOe. Inset: Plot of ln(*χ*_M_′*T*) *vs T*^−1^.

Variable-temperature magnetic susceptibility data for compounds **1** and **2** were measured in the temperature range of 1.8–300 K in a direct current (dc) field of 2 kOe. The *χ*_M_*T* vs *T* plots of **1** and **2** are shown in Fig. 2 and Supplementary Fig. S3. At 300 K, the *χ*_M_*T* values of 3.35 cm^3^ K mol^**−**1^ (**1**) and 3.45 cm^3^ K mol^**−**1^ (**2**) are much higher than the spin-only value of 2.25 cm^3^ K mol^**−**1^ for one isolated W^V^ center (*S* = 1/2) and one isolated high-spin Co^II^ center (*S* = 3/2) with *g* = 2.00, indicating the significant orbital contribution of high-spin Co^II^ in an octahedral configuration. Upon cooling, *χ*_M_*T* increases continuously to a maximum value of 73.17 cm^3^ mol^−1^ K (**1**) and 73.14 cm^3^ mol^−1^ K (**2**) at 14 K, before dropping quickly at lower temperatures. The increase in *χ*_M_*T* with decreasing temperature indicates the intrachain ferromagnetic coupling between W^V^ and Co^II^ centers, which has been reported in other Co^II^-W^V^ systems^43^,^44^,^45^,^46^,^47^. The further sharp decrease below 14 K is attributed to a saturation of the *χ*_M_ value and/or zero-field splitting (ZFS) effect, which urged us to explore the possibility of magnetic blocking. The field-cooled (FC) and zero-field-cooled (ZFC) magnetization was thus measured at *H*_dc_ = 10 Oe as shown in Supplementary Figs S4 and S5. The ZFC and FC curves diverge at 10.2 and 8.4 K for compounds **1** and **2**, which define the blocking temperatures of these chain compounds.

**Figure 3:**
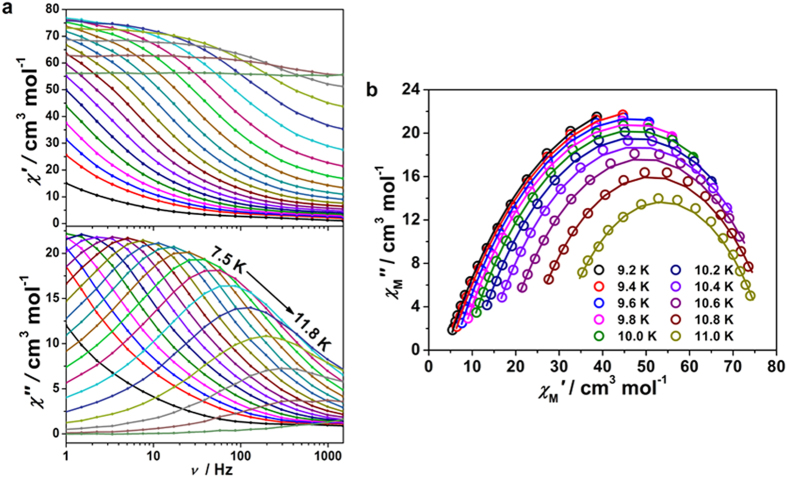
The ac susceptibility and Cole-Cole diagram for 1. (**a**) Temperature dependence of in-phase (top) and out-of-phase (bottom) components of the ac susceptibility for **1** in zero applied static field with a 1Oe oscillating field at a frequency of 1–1500 Hz; (**b**) Cole-Cole diagram of **1**, plotted using *χ*_M_′ and *χ*_M_′′ at different temperature. The solid lines represent the fits to a general Debye model.

To verify the dynamics of the magnetization relaxation, ac magnetic measurements were performed on polycrystalline samples of **1** and **2** under a zero dc field with *H*_ac_ = 1 Oe in the frequency range of 1–1500 Hz (Fig. 3a, Supplementary Figs S6a and S7). Below 12 K, the obvious frequency dependence of both in-phase (*χ*_M_′) and out-of-phase (*χ*_M_′′) ac susceptibility was observed as typically observed in SCMs. The Mydosh parameter *φ* = (Δ*T*/*T*)/Δ(log *f*_p_) (where *f*_p_ is the frequency at which a maximum appears in the *χ*_M_′′(*f*) plot^12^,^48^,^49^, was estimated to be ca 0.13, which falls into the category of superparamagnets (either SMMs or SCMs) and considerably greater than that for a spin glass^50^,^51^. Notably, both the peaks of the *χ*_M_′ and *χ*_M_′′ signals are rather broad, indicating the existence of at least two possible relaxation processes. Futhermore, the frequency dependent ac data also show pronounced temperature dependence, from which the semicircular Cole-Cole plots (*χ*_M_′′ vs. *χ*_M_′) were obtained (Fig. 3b and Supplementary Fig. S6b). A generalized Debye mode^52^ was used to extract the values and distribution of the relaxation time *τ* (Supplementary Tables 5 and 6). The obtained α values are 0.35–0.41 and 0.40–0.42 for **1** and **2**, respectively. These values indicated a relatively wide distribution of relaxation times, which might be caused by the variable distributions in chain length and/or interchain magnetic interactions and/or random defects as found in other cases where α ranges from 0 to 0.7^53^.

For an Ising-like or anisotropic Heisenberg one-dimensional system, the *χ*_M_*T* value in zero applied field is directly proportional to the correlation length *ξ*. Therefore, the *χ*_M_*T* increases exponentially with lowering temperature, following the equation: *χ*_M_*T *≈ *C*_eff_ × exp (Δ_ξ_/*k*_B_*T*)^18^,^20^,^54^, where *C*_eff_ is the effective Curie constant, and Δ_ξ_ gives an estimation of the intrachain exchange energy cost to create a domain wall along the chain. Thus, provided the 1D nature of compounds **1** and **2** and the presence of significant anisotropy, plots of ln (*χ*_M_′*T*) versus 1/*T* of both compounds should display a linear region, which is actually observed experimentally. As can be seen in the inset of Fig. 2 and Supplementary Fig. S3, the ln(*χ*_M_′*T*) versus *T*^−1^ plots measured at *H*_dc_ = 0 Oe and *H*_ac_ = 1 Oe at a frequency of 1 Hz feature a linear region in the temperature range of 11.6–30 K for both **1** and **2**, yielding Δ_ξ_/k_B_ = 78(0) K and *C*_eff_ = 0.83(1) cm^3^ K mol^**−**1^ for **1**, and Δ_ξ_/k_B_ = 78 (0) K and *C*_eff_ = 0.88 (1) cm^3^ K mol^**−**1^ for **2**, respectively. Below 11.6 K, ln (*χ*_M_′*T*) reaches a maximum and then undergoes a linear decrease with decreasing temperature, which indicates that the correlation length becomes larger than the average distance between two intrinsic defects along the chain, as the interchain interactions are not likely for these well isolated 1D chain^19^,^55^. From the activation energy Δ_ξ_, the exchange interaction *J* in the Ising limit can be estimated through the expression Δ_ξ_ = 4|*J*|*S*_eff_^2^,^54^,^55^,^56^. Assuming the system at low temperature to be an Ising chain with the effective spin approach *S*_Co, eff_ = 1/2 and *S*_W_ = 1/2, the intrachain interaction is calculated as *J*/*k*_B_ = 19.5 K for both **1** and **2**. As the Δ_ξ_ depends not only on the exchange interaction (*J*) but also the single-ion anisotropy, the *J* value calculated from the Ising model is a rough estimation.

**Figure 4:**
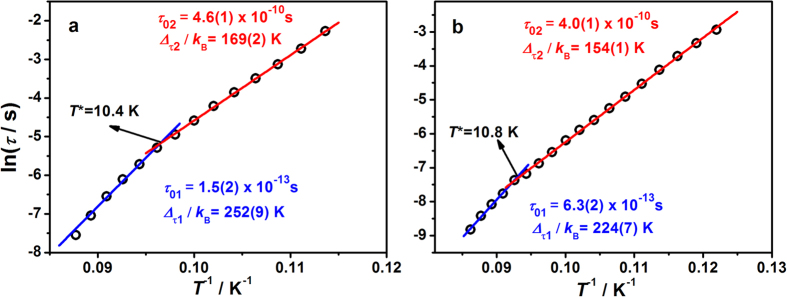
Arrhenius plots of relaxation times for 1 **(a)** and 2 **(b)**. The solid lines show linear fits of the experimental data according to the Arrhenius law.

The Glauber dynamics predicts that the energy barrier for the spin reversal of an ideal Ising chain should be Δ_τ_ = 2Δ_ξ_ for infinite chains and Δ_τ_ = Δ_ξ_ for finite-size chains, where growth of the correlation length is limited by naturally occurring defects^57^,^58^. However, for most real SCMs, an anisotropic Heisenberg chain model is more suitable, which takes into account the magnetic anisotropy energy of each magnetic unit^59^. As the correlation length *ξ* increases exponentially with a decrease in temperature, the overall energy barrier Δ_τ_ is Δ_τ_ = 2Δ_ξ_ + Δ_A_ for the infinite chain at high temperature and Δ_τ_ = Δ_ξ_ + Δ_A_ for the finite-size chain at low temperature, where Δ_A_ represents the intrinsic anisotropic barrier for the individual spin in the absence of magnetic exchange. This is actually the case for **1** and **2**. From the Arrhenius plots of relaxation times *τ* obtained from the frequency-dependent *χ*_M_′′ peaks, two thermally activated regions were obviously observed above and below the crossover temperatures of T* = 10.4 and 10.8 K for **1** and **2**, respectively. These two regions are corresponding to the infinite-size and finite-size regimes of relaxation, commonly encountered for SCMs. The energy barriers for both of these thermally activated processes were estimated using the Arrhenius law *τ* = *τ*_0_ exp (Δ_τ_/*k*_B_*T*), giving the Δ_τ1_ (*τ*_01_) and Δ_τ2_ (*τ*_01_) as 252(9) K (1.5(2) × 10^**−**13 ^s) and 169(2) K (4.6(1) × 10^**−**10^ s) for **1** and 224(7) K (6.3(2) × 10^**−**13^ s) and 154(1) K (4.0(1) × 10^**−**10^ s) for **2**, respectively (Fig. 4). To ensure the integrity of data, all the peak values of compound **1** (Fig. 4a) were read from the data of ac susceptibility (Fig. 3a). However, as the peak of 11.4 K is too broad to read accurately, the first spot was excluded in the fitting process. Remarkably, the barriers of 252(9) and 224(7) K observed for **1** and **2** are among the highest for single-chain magnets. The radical bridged chain compounds, [Co(hfac)_2_PyNN]_n_^40^ and [Co(hfac)_2_NaphNN]_n_^41^, were reported to display a relaxation barrier of (396 ± 13) K and (398 ± 14) K, respectively. As such, to the best of our knowledge, the energy barriers of compounds **1** and **2** set the new records for cyano-bridged SCMs and are preceded only by these two Co^II^-radical compounds among all SCMs. Obviously, for **1** and **2** in both the infinite-size and the finite-size regimes, Δ_τ1_ is larger than 2Δ_ξ_ and Δ_τ2_ is larger than Δ_ξ_. From these values, the intrinsic anisotropic barrier Δ_A_ can be estimated either by Δ_A_ = Δ_τ1_ − 2Δ_ξ_ or Δ_A_ = Δ_τ2_ − Δ_ξ_. The obtained values are 96 or 91 K for **1**, and 68 or 76 K for **2**, respectively.

**Figure 5:**
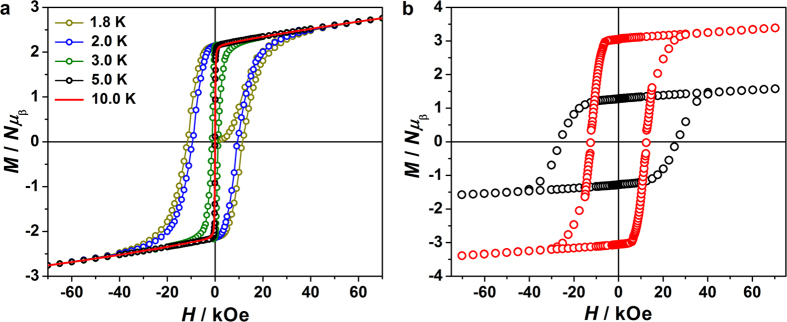
The dc variable-field magnetization of 1. (**a**) Magnetic hysteresis loops of polycrystalline **1** measured at 1.8, 2, 3, 5 and 10 K with a field sweep rate of 500 Oe/s. Solid line is guide for eyes; (**b**) Hysteresis loops at 1.8 K on the oriented long crystal bundle of **1** along (black) and perpendicular (red) to the chain direction (*a* axis).

**Figure 6:**
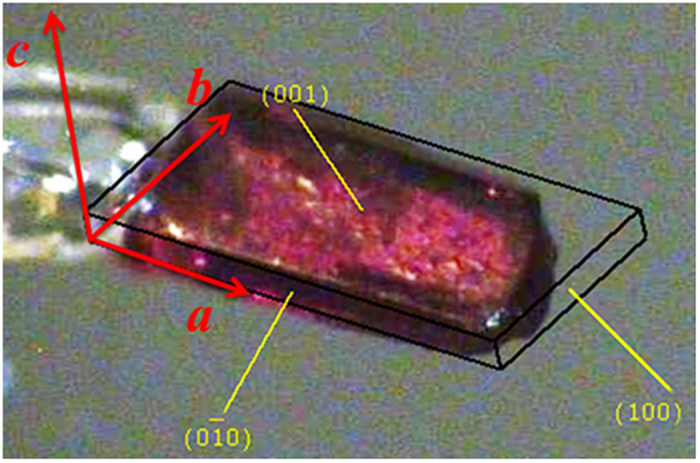
Face index of single crystal of 1.

Furthermore, the slow magnetic relaxation of both **1** and **2** were confirmed by the observation of the magnetization hysteresis loops. The loops were firstly measured on the polycrystalline samples at different temperatures and depicted in Fig. 5a and Supplementary Fig. S8a. At 1.8 K, the largest magnetization values at 70 kOe, 2.76 *Nμ*_B_ (**1**) and 2.75 *Nμ*_B_ (**2**), are significantly lower than the estimated saturation value of 3.2 *Nμ*_B_ for the effective spin approach *S*_Co,eff_ = 1/2, *g*_Co_ = 13/3^60^,^61^, and *g*_W_ = 2, *S*_W_ = 1/2 indicating the significant magnetic anisotropy^34^,^49^,^54^. Obvious hysteresis loops can be observed below 5 K, with the coercive field of 11.5 kOe for **1** and 9.9 kOe for **2** at 1.8 K. Fortunately, although the single crystals of **1** and **2** are not big enough for the magnetic measurement on one single crystal, these strip-like single crystals grow parallel and form a bundle of about 1.0 × 1.5 × 6.5 mm (Supplementary Fig. S9), which is suitable for the anisotropic measurement. By the face index of the crystal (Fig. 6), we can see that the long edge of the crystal strip (and thus the long side of the crystal bundle) is along the *a* axis, which is parallel to the direction of the 3, 3-ladder. Thus, the anisotropic magnetization measurements were performed with the dc field applied parallel or perpendicular to the direction of the 3, 3-ladder. As can be seen from the loops at 1.8 K (Fig. 5b and Supplementary Fig. S8b), strong magnetic anisotropy of both compounds is reflected incisively and vividly. When the field is applied along the chain direction, the saturated magnetization value is much lower and exhibits a very large hysteresis loop with a coercive field of *H*_c_ = 26.2 kOe for **1** (22.6 kOe for **2**) and a remnant magnetization of *M*_r_ = 1.28 *Nμ*_B_ for **1** (1.27 *Nμ*_B_ for **2**) at 1.8 K. With the field being perpendicular to the chain direction however, the compounds are quite easily magnetized. At 1.8 K, *M* reaches 3.4 *Nμ*_B_ at 70 kOe (3.9 *Nμ*_B_ for **2**) with *H*_c_ = 12.5 kOe for **1** (7.9 kOe for **2**) and *M*_r_ = 3.1 *Nμ*_B_ for **1** (3.6 *Nμ*_B_ for **2**). This behavior is consistent with the ferromagnetic coupling between Co^II^ and W^V^ centers^43^,^60^,^61^,^62^. The observation of the loops clearly shows the substantial slow magnetic relaxation, indicative of a “magnetic memory” of both compounds **1** and **2**.

In summary, two unique cryano-bridged 3, 3-ladder were synthesized from the anisotropic Co^II^ center, the octacyanotungsten, and two bulky counter cations. Because of the intrachain ferromagnetic interaction, strong magnetic anisotropy, as well as the well isolation of individual chains, these 1D compounds behave as single-chain magnets with record high spin reversal barriers (252(9) and 224(7) K) for the cyano-bridged SCMs. Furthermore, hysteresis loops can be observed for both compounds below 5 K with large coercivities of 26.2 and 22.6 kOe at 1.8 K. Efforts to extend this synthetic strategy towards other SCMs using the 4d/5d cyanometallates and other anisotropic metal centers with higher energy barriers and blocking temperatures are underway.

## Methods

### Materials

All of the reagents and solvents were purchased from commercial sources and used as received. The complex Cs_3_[W(CN)_8_]·2H_2_O was prepared by reported procedures^63^,^64^.

Caution! Although no problems were encountered in the preparation of the following complexes, in the acidic reaction conditions, suitable precautions should be taken when handling potentially poisonous compounds. It is of the utmost importance that all preparations should be performed and stored in well-ventilated areas.

### Synthesis of 1

Single crystals of **1** were achieved by the slow diffusion of the reactants in the presence of the bulky Ph_4_P^+^ and Ph_4_As^+^ cations in H-shaped tube. 3-methylpyridine (0.5 mmol, 46.5 mg) was added dropwise into a stirred aqueous solution (2.5 mL) of CoCl_2_·6H_2_O (0.25 mmol, 59 mg), and Ph_4_PCl (0.25 mmol, 94 mg) was added after 30 minutes. Then the solution was carefully added to one side of an H-shaped tube. The other arm contained an aqueous solution (2.5 mL) of Cs_3_[W(CN)_8_]·2H_2_O (0.125 mmol, 103.5 mg). A methanol-water (v/v, 1:3) mixture was used as a buffer between the two arms. The dark red strip single crystals suitable for X-ray diffraction of **1** were obtained. Elemental analysis (%): Calcd. for C_48.2_H_40.7_N_10.7_O_0.9_CoWP: C 54.72, H 3.88, N 14.17; found: C 54.45, H 4.10, N 14.33%. IR: *ν*(RO–H) 3407.7(w), *ν*(C–H_py_) 3026.8(w), 1107.5(vs), *ν*(C–H_Me_) 2923.1(w), *ν*(C≡N) 2146.0(w), *ν*(C=C, C=N) 1583.4(m), 1483.5(s), 1439.5(s).

### Synthesis of 2

The synthesis of compound **2** is analogous to that of **1** but using Ph_4_AsCl (0.25 mmol, 105 mg) instead of Ph_4_PCl. The dark red strip block crystals of **2** suitable for single-crystal X-ray analysis were obtained after 4 weeks. Elemental analysis (%): Calc. for C_50_H_41_N_11_CoWAs (**2**): C 53.93, H 3.71, N 13.84. Found: C, 54.09; H, 3.82; N, 13.43. IR: *ν*(RO–H) 3411.3(w), *ν*(C–H_py_) 3081.1(w), 1109.2(vs), *ν*(C–H_Me_) 2922.6(w), *ν*(C≡N) 2145.6(w), *ν*(C=C, C=N) 1579.4(s), 1482.9(s), 1439.7(vs).

### X-ray Crystallography

Crystallographic data of compounds **1** and **2** were collected on Photon100 CMOS detector and Bruker Smart CCD area-detector diffractometer with *Mo-Kα* radiation (*λ* = 0.71073 Å) by using an *ω* scan mode at 123 K and 296 K, respectively. The diffraction data were treated using SAINT^65^, and all absorption corrections were applied by using SADABS^66^. All non-hydrogen atoms were located by Patterson method^67^ using the SHELXS programs of the SHELXTL package and subsequent difference Fourier syntheses. Hydrogen bonded to carbon were determined theoretically and refined with isotropic thermal parameters riding on their parents. H-atoms of water and methanol were first located by difference Fourier E-maps and then treated isotropically as riding. All non-hydrogen atoms were refined by full-matrix least-squares on *F*^2^. All calculations were performed by SHELXTL-97^68^,^69^.

### Measurements

The IR spectra were carried out with a Nexus 870 FT-IR spectrometer using KBr pellets in the range of 400–4000 cm^**−1**^. Elemental analyses of C, H, N were recorded on a PerkinElmer 240 C elemental analyzer. The temperature-dependent magnetic susceptibility, the zero-field cooled magnetization and field-cooled magnetization, the anisotropy measurement at 1.8 K and AC magnetic susceptibility were measured using a Quantum Design MPMS-XL7 superconducting quantum interference device (SQUID) magnetometer. Magnetization hysteresis loop at 1.8–10 K from 7 to −7 T and back were measured using an eicosane-constrained sample to prevent sample torquing on a Quantum Design VSM SQUID magnetometer. Experimental susceptibilities were corrected for the diamagnetism estimated Pascal′s tables and for sample holder by previous calibration.

## Additional Information

**Accession codes:** Crystallographic data for the structural analysis of the compounds have been deposited with the Cambridge Crystallographic Data Centre, under CCDC no. 1419479 (**1**) and 1419480 (**2**). These data can be obtained free of charge from The Cambridge Crystallographic Data Centre via www. ccdc.cam.ac.uk/data_request/cif.

**How to cite this article**: Wei, R.-M. *et al.* Single-Chain Magnets Based on Octacyanotungstate with the Highest Energy Barriers for Cyanide Compounds. *Sci. Rep.* **6**, 24372; doi: 10.1038/srep24372 (2016).

## Supplementary Material

Supplementary Information

## References

[b1] Glauber R. J. Time-dependent statistics of the ising model. J. Math. Phy. 4, 294–307 (1963).

[b2] Caneschi A. *et al.* Cobalt(II)-nitronyl nitroxide chains as molecular magnetic nanowires. Angew. Chem. Int. Ed. 40, 1760–1763 (2001). 10.1002/1521-3773(20010504)40:9<1760::aid-anie17600>3.0.co;2-u 11353503

[b3] Clérac R., Miyasaka H., Yamashita M. & Coulon C. Evidence for single-chain magnet behavior in a Mn^III^−Ni^II^ chain designed with high spin magnetic units: a route to high temperature metastable magnets. J. Am. Chem. Soc. 124, 12837–12844 (2002). 12392430 10.1021/ja0203115

[b4] Guo J.-F. *et al.* One-dimensional ferromagnetically coupled bimetallic chains constructed with trans-[Ru(acac)_2_(CN)_2_]^−^: syntheses, structures, magnetic properties, and density functional theoretical study. Chem. Eur. J. 16, 3524–3535 (2010). 20140917 10.1002/chem.200902047

[b5] Feng X., David Harris T. & Long J. R. Influence of structure on exchange strength and relaxation barrier in a series of Fe^II^Re^IV^(CN)_2_ single-chain magnets. Chem. Sci. 2, 1688–1694 (2011).

[b6] Liu T.-F. *et al.* An azide-bridged homospin single-chain magnet: [Co(2, 2′-bithiazoline)(N_3_)_2_]_n_. J. Am. Chem. Soc. 125, 13976–13977 (2003). 14611231 10.1021/ja0380751

[b7] Pardo E. *et al.* Cobalt(II)-copper(II) bimetallic chains as a new class of single-chain magnets. Adv. Mater. 16, 1597–1600 (2004).

[b8] Bernot K., Bogani L., Caneschi A., Gatteschi D. & Sessoli R. A family of rare-earth-based single chain magnets: playing with anisotropy. J. Am. Chem. Soc. 128, 7947–7956 (2006). 16771509 10.1021/ja061101l

[b9] Xu H.-B., Wang B.-W., Pan F., Wang Z.-M. & Gao S. Stringing oxo-centered trinuclear [Mn^III^_3_O] units into single-chain magnets with formate or azide linkers. Angew. Chem. Int. Ed. 46, 7388–7392 (2007). 10.1002/anie.200702648 17705325

[b10] Coronado E., Galán-Mascarós J. R. & Martí-Gastaldo C. Single chain magnets based on the oxalate ligand. J. Am. Chem. Soc. 130, 14987–14989 (2008). 18937480 10.1021/ja806298t

[b11] Hoshino N. *et al.* Three-way switching in a cyanide-bridged [FeCo] chain. Nat. Chem. 4, 921–926 (2012). 23089867 10.1038/nchem.1455

[b12] Mougel V. *et al.* A uranium-based UO^2+^–Mn^2+^ single-chain magnet assembled trough cation–cation interactions. Angew. Chem. Int. Ed. 53, 819–823 (2014). 10.1002/anie.201307366 PMC4232274 24311434

[b13] Yoon J. H. *et al.* An end-on azide-bridged antiferromagnetic single-chain magnet involving spin canting and field-induced two-step magnetic transitions. Chem. Eur. J. 15, 3661–3665 (2009). 19274695 10.1002/chem.200900250

[b14] Yoon J. H. *et al.* One-dimensional end-to-end azide-bridged Mn^III^ complexes incorporating alkali metal ions: slow magnetic relaxations and metamagnetism. Chem. Eur. J. 17, 3028–3034 (2011). 21284046 10.1002/chem.201002269

[b15] Ferbinteanu M. *et al.* Single-chain magnet (NET_4_)Mn_2_(5-MeOsalen)_2_Fe(CN)_6_ made of Mn^III^–Fe^III^–Mn^III^ trinuclear single-molecule magnet with an *S*t = 9/2 spin ground state. J. Am. Chem. Soc. 127, 3090–3099 (2005). 15740148 10.1021/ja0468123

[b16] Toma L. M. *et al.* Fe(bpym)(CN)_4_^−^: A new building block for designing single-chain magnets. J. Am. Chem. Soc. 128, 4842–4853 (2006). 16594721 10.1021/ja058030v

[b17] Visinescu D. *et al.* First heterotrimetallic {3d-4d-4f} single chain magnet, constructed from anisotropic high-spin heterometallic nodes and paramagnetic spacers. Chem. Eur. J. 15, 11808–11814 (2009). 19806623 10.1002/chem.200902408

[b18] Miyasaka H. *et al.* Cyano-bridged Mn^III^–M^III^ single-chain magnets with M^III^ = Co^III^, Fe^III^, Mn^III^, and Cr^III^. Chem. Eur. J. 18, 3942–3954 (2012). 22344962 10.1002/chem.201102738

[b19] Rams M., Peresypkina E. V., Mironov V. S., Wernsdorfer W. & Vostrikova K. E. Magnetic relaxation of 1D coordination polymers (X)_2_Mn(acacen)Fe(CN)_6_, X = Ph_4_P^+^, Et_4_N^+^. Inorg. Chem. 53, 10291–10300 (2014). 25219567 10.1021/ic501330j

[b20] Zhang Y.-Z., Zhao H.-H., Funck E. & Dunbar K. R. A single-chain magnet tape based on hexacyanomanganate(III). Angew. Chem. Int. Ed. 54, 5583–5587 (2015). 10.1002/anie.201410664 25784624

[b21] Lescouëzec R. *et al.* Cyanide-bridged iron(III)–cobalt(II) double zigzag ferromagnetic chains: two new molecular magnetic nanowires. Angew. Chem. Int. Ed. 42, 1483–1486 (2003). 10.1002/anie.200250243 12698479

[b22] Wen H.-R. *et al.* Synthesis, crystal structures, and magnetic properties of cyano-bridged heterobimetallic chains based on [(Tp)Fe(CN)_3_]. Inorg. Chem. 45, 8942–8949 (2006). 17054353 10.1021/ic060928d

[b23] Choi S. W. *et al.* One-dimensional cyanide-bridged Mn^III^W^V^ bimetallic complexes: metamagnetism, spontaneous resolution, and slow magnetic relaxation. Inorg. Chem. 48, 9066–9068 (2009). 19739638 10.1021/ic901610j

[b24] Dong D.-P. *et al.* Photoswitchable dynamic magnetic relaxation in a well-isolated {Fe_2_Co} double-zigzag chain. Angew. Chem. Int. Ed. 51, 5119–5123 (2012). 10.1002/anie.201105987 22499533

[b25] Liu T. *et al.* A light-induced spin crossover actuated single-chain magnet. Nat. Commun. 4, 2826–2832 (2013).

[b26] Herrera J. M. *et al.* Reversible photoinduced magnetic properties in the heptanuclear complex [Mo^IV^(CN)_2_(CN–CuL)_6_]^8+^: a photomagnetic high-spin molecule. Angew. Chem. Int. Ed. 43, 5468–5471 (2004). 10.1002/anie.200460387 15372638

[b27] Wang Z. X. *et al.* A sodalite-like framework based on octacyanomolybdate and neodymium with guest methanol molecules and neodymium octahydrate ions. Angew. Chem. Int. Ed. 45, 3287–3291 (2006). 10.1002/anie.200600455 16607666

[b28] Arai M., Kosaka W., Matsuda T. & Ohkoshi S. Observation of an iron(II) spin-crossover in an iron octacyanoniobate-based magnet. Angew. Chem. Int. Ed. 47, 6885–6887 (2008). 10.1002/anie.200802266 18683172

[b29] Nowicka B. *et al.* The impact of ligands upon topology and functionality of octacyanidometallate-based assemblies. Coord. Chem. Rev. 256, 1946–1971 (2012).

[b30] Pinkowicz D. *et al.* Enforcing multifunctionality: a pressure-induced spin-crossover photomagnet. J. Am. Chem. Soc. 137, 8795–8802 (2015). 26098129 10.1021/jacs.5b04303

[b31] Song Y. *et al.* Synthesis, crystal structures, and magnetic properties of two cyano-bridged tungstate(V)−manganese(II) bimetallic magnets. Inorg. Chem. 42, 1848–1856 (2003). 12639117 10.1021/ic025959x

[b32] Venkatakrishnan T. S. *et al.* Enhanced ion anisotropy by nonconventional coordination geometry: single-chain magnet behavior for a [{Fe^II^L}_2_{Nb^IV^(CN)_8_}] helical chain compound design with heptacoordinate Fe^II^. J. Am. Chem. Soc. 132, 6047–6056 (2010). 20380425 10.1021/ja9089389

[b33] Chorazy S. *et al.* Conjunction of chirality and slow magnetic relaxation in the supramolecular network constructed of crossed cyano-bridged Co^II^–W^V^ molecular chains. J. Am. Chem. Soc. 134, 16151–16154 (2012). 22989141 10.1021/ja307520k

[b34] Miyasaka H. *et al.* Three-dimensional antiferromagnetic order of single-chain magnets: a new approach to design molecule-based magnets. Chem. Eur. J. 16, 3656–3662 (2010). 20151436 10.1002/chem.200902861

[b35] Coulon C., Clérac R., Wernsdorfer W. Colin T. & Miyasaka H. Realization of a magnet using an antiferromagnetic phase of single-chain magnets. Phys. Rev. Lett. 102, 167204 (2009). 19518751 10.1103/PhysRevLett.102.167204

[b36] Miyasaka H. *et al.* [Mn_2_(saltmen)_2_Ni(pao)_2_(L)_2_](A)_2_ with L = pyridine, 4-picoline, 4-*tert*-Butylpyridine, *N*-Methylimidazole and A = ClO_4_^–^, BF_4_^–^, PF_6_^–^, ReO_4_^–^: a family of single-chain magnets. Inorg. Chem. 42, 8203–8213 (2003). 14658870 10.1021/ic034872o

[b37] Miyasaka H., Saitoh A., Yamashita M. & Clérac R. A Mn^III^_2_Ni^II^ single-chain magnet separated by a thick isolating network of BPh_4_^–^ anions. Dalton Trans. 2422–2427 (2008). 18461197 10.1039/b718036e

[b38] Song Y. *et al.* Octacyanometallate-based single-molecule magnets: Co^II^_9_M^V^_6_ (M = W, Mo). J. Am. Chem. Soc. 127, 3708–3709 (2005). 15771500 10.1021/ja042334k

[b39] Černák J. *et al.* Cyanocomplexes with one-dimensional structures: preparations, crystal structures and magnetic properties. Coord. Chem. Rev. 224, 51–66 (2002).

[b40] Vaz M. G. *et al.* A cobalt pyrenylnitronylnitroxide single-chain magnet with high coercivity and record blocking temperature. Chem. Eur. J. 20, 5460–5467 (2014). 24644094 10.1002/chem.201304852

[b41] Cassaro R. A. A. *et al.* A single-chain magnet with a very high blocking temperature and a strong coercive field. Inorg. Chem. 54, 9381–9383 (2015). 26366631 10.1021/acs.inorgchem.5b01431

[b42] Llunell M., Casanova D., Cirera J., Alemany P. & Alvarez S. R: *SHAPE, Version 2.1*. Universitat de Barcelona, Barcelona, Spain. URL http://www.ee.ub.edu/ (2013).

[b43] Arimoto Y. *et al.* Photoinduced magnetization in a two-dimensional cobalt octacyanotungstate. J. Am. Chem. Soc. 125, 9240–9241 (2003). 12889922 10.1021/ja030130i

[b44] Herrera J. M. *et al.* Crystal structures and magnetic properties of two octacyanotungstate(IV) and (V)-cobalt(II) three-dimensional bimetallic frameworks. Inorg. Chem. 42, 7052–7059 (2003). 14577772 10.1021/ic034188+

[b45] Clima S. *et al.* Effect of the metal environment on the ferromagnetic interaction in the Co−NC−W pairs of octacyanotungstate(V)−cobalt(II) three-dimensional networks. Inorg. Chem. 46, 2682–2690 (2007). 17348645 10.1021/ic062345+

[b46] Wang J. *et al.* Octacyanotungstate(V)-based square W_2_M_2_ (M = Co, Mn) complexes: synthesis, structure and magnetic properties. Dalton Trans. 39, 3489–3494 (2010). 20333337 10.1039/b924203a

[b47] Ozaki N. *et al.* Photoinduced magnetization with a high curie temperature and a large coercive field in a Co-W bimetallic assembly. Adv. Funct. Mater. 22, 2089–2093 (2012).

[b48] Wang S. *et al.* The observation of superparamagnetic behavior in molecular nanowires. J. Am. Chem. Soc. 126, 8900–8901 (2004). 15264813 10.1021/ja0483995

[b49] Zhang J.-Y., Wang K., Li X.-B. & Gao E.-Q. Magnetic coupling and slow relaxation of magnetization in chain-based Mn^II^, Co^II^, and Ni^II^ coordination frameworks. Inorg. Chem. 53, 9306–9314 (2014). 25136992 10.1021/ic5014279

[b50] Miller J. S. Magnetically ordered molecule-based materials. Chem. Soc. Rev. 40, 3266–3296 (2011). 21479292 10.1039/c0cs00166j

[b51] Mydosh J. A. In Spin Glasses: An Experimental Introduction Ch. 3, 64–72 (Taylor & Francis: London, 1993).

[b52] Guo Y.-N., Xu G.-F., Guo Y. & Tang J. Relaxation dynamics of dysprosium(III) single molecule magnets. Dalton Trans. 40, 9953–9963 (2011). 21829790 10.1039/c1dt10474h

[b53] Sun H.-L., Wang Z.-M. & Gao S. Strategies towards single-chain magnets. Coord. Chem. Rev. 254, 1081–1100 (2010).

[b54] Coulon C., Miyasaka H. & Clérac R. Single-chain magnets: theoretical approach and experimental systems. Struct. Bonding (Berlin) 122, 163–206 (2006).

[b55] Harris T. D., Bennett M. V., Clérac R. & Long J. R. [ReCl_4_(CN)_2_]^2−^: a high magnetic anisotropy building unit giving rise to the single-chain magnets (DMF)_4_MReCl_4_(CN)_2_ (M = Mn, Fe, Co, Ni). J. Am. Chem. Soc. 132, 3980–3988 (2010). 20192267 10.1021/ja910963x

[b56] Miyasaka H., Julve M., Yamashita M. & Clérac R. Slow dynamics of the magnetization in one-dimensional coordination polymers: single-chain magnets. Inorg. Chem. 48, 3420–3437 (2009). 19361243 10.1021/ic802050j

[b57] Bogani L. *et al.* Finite-size effects in single chain magnets: an experimental and theoretical study. Phys. Rev. Lett. 92, 207204 (2004). 15169379 10.1103/PhysRevLett.92.207204

[b58] Bogani L., Vindigni A., Sessoli R. & Gatteschi D. Single chain magnets: where to from here? J. Mater. Chem. 18, 4750–4758 (2008).

[b59] Zhang W.-X., Ishikawa R., Breedlove B. & Yamashita M. Single-chain magnets: beyond the glauber model. RSC Adv. 3, 3772–3798 (2013).

[b60] Ohkoshi S., Ikeda S., Hozumi T., Kashiwagi T. & Hashimoto K. Photoinduced magnetization with a high curie temperature and a large coercive field in a cyano-bridged cobalt−tungstate bimetallic assembly. J. Am. Chem. Soc. 128, 5320–5321 (2006). 16620085 10.1021/ja060510e

[b61] Ohkoshi S., Hamada Y., Matsuda T., Tsunobuchi Y. & Tokoro H. Crystal structure, charge-transfer-induced spin transition, and photoreversible magnetism in a cyano-bridged cobalt−tungstate bimetallic assembly. Chem. Mater. 20, 3048–3054 (2008).

[b62] Nakabayashi K. *et al.* Cesium cyano-bridged Co^II^–M^V^ (M = Mo and W) layered frameworks exhibiting high thermal durability and metamagnetism. Crys. Growth Des. 14, 6093–6100 (2014).

[b63] Leipoldt J. G., Bok L. D. C. & Cilliers P. J. The preparation of potassium octacyanotungstate(IV) dihydrate. Z. Anorg. Allg. Chem. 407, 350–352 (1974).

[b64] Bok L. D. C., Leipoldt J. G. & Basson S. S. The preparation of Cs_3_Mo(CN)_8_·2H_2_O and Cs_3_W(CN)_8_·2H_2_O. Z. Anorg. Allg. Chem. 415, 81–83 (1975).

[b65] *SAINT v5.0–6.01,* Bruker Analytical X-ray Systems Inc.: Madison, WI (1998).

[b66] Krause L., Herbst-Irmer R., Sheldrick G. M. & Stalke D. Comparison of silver and molybdenum microfocus X-ray sources for single-crystal structure determination. J. Appl. Cryst. 48, 3–10 (2015). 26089746 10.1107/S1600576714022985 PMC4453166

[b67] Patterson A. L. A fourier series method for the determination of the components of interatomic distances in crystals. Phys. Rev. 46, 372–376 (1934).

[b68] Sheldrick G. M. SHELXT-Integrated space-group and crystal-structure determination. Acta Cryst. A71, 3–8 (2015). 10.1107/S2053273314026370 PMC4283466 25537383

[b69] Sheldrick G. M. Crystal structure refinement with SHELXL. Acta Cryst. C71, 3–8 (2015). 10.1107/S2053229614024218 PMC4294323 25567568

