# Supplementary material for: Single-Chain Magnets Based on Octacyanotungstate with the Highest Energy Barriers for Cyanide Compounds
Source: Sci Rep. 2016 Apr 13;6:24372. doi: 10.1038/srep24372 (PMC4829875; doi:10.1038/srep24372)
Supplement: Supplementary Information [file srep24372-s1.doc]

**Supplementary Information**

**Single-Chain Magnets Based on Octacyanotungstate with the Highest Energy Barriers for Cyanide Compounds**

Rong-Min Wei,^1,2^ Fan Cao,^1^ Jing Li,^1^ Li Yang,^1^ Yuan Han,^1^ Xiu-Ling Zhang,^2^ Zaichao Zhang,*^3^ Xin-Yi Wang,*^1^ & You Song*^1^

^1^ State Key Laboratory of Coordinate Chemistry, Collaborative Innovation Center of Advanced Microstructures, School of Chemistry and Chemical Engineering, Nanjing University, Nanjing 210093, China. ^2^ Key Laboratory of Coordination Chemistry and Functional Materials in Universities of Shandong, School of Chemistry and Chemical Engineering, Dezhou University, Dezhou 253023, China. ^3^ Jiangsu Key Laboratory for the Chemistry of Low-Dimensional Materials, School of Chemistry and Chemical Engineering, Huaiyin Normal University, Huai’an 223300, China. Correspondence and requests for materials should be addressed to Y.S. (email: yousong@nju.edu.cn), X.-Y.W. (email: wangxy66@nju.edu.cn) or Z.Z. (email: zhangzc@hytc.edu.cn).


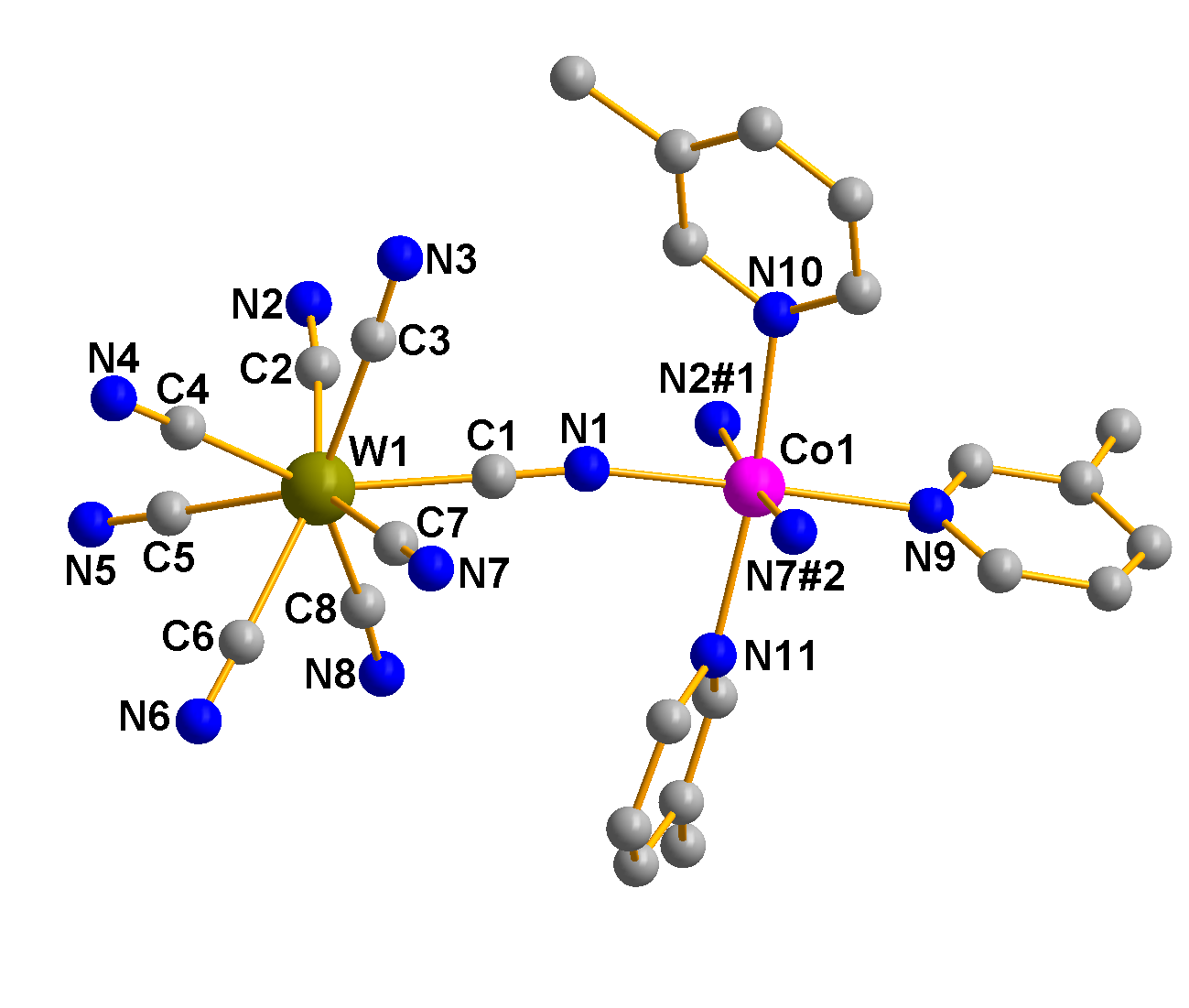


**Supplementary Figure S1.** The asymmetric unit of compound **2**; Hydrogen atoms and Ph_4_As^+^ cations were omitted for clarity. Symmetry code: #1=2-*x*, 2-*y*, 1-*z*; #2=1-*x*, 2-*y*, 1-*z*.


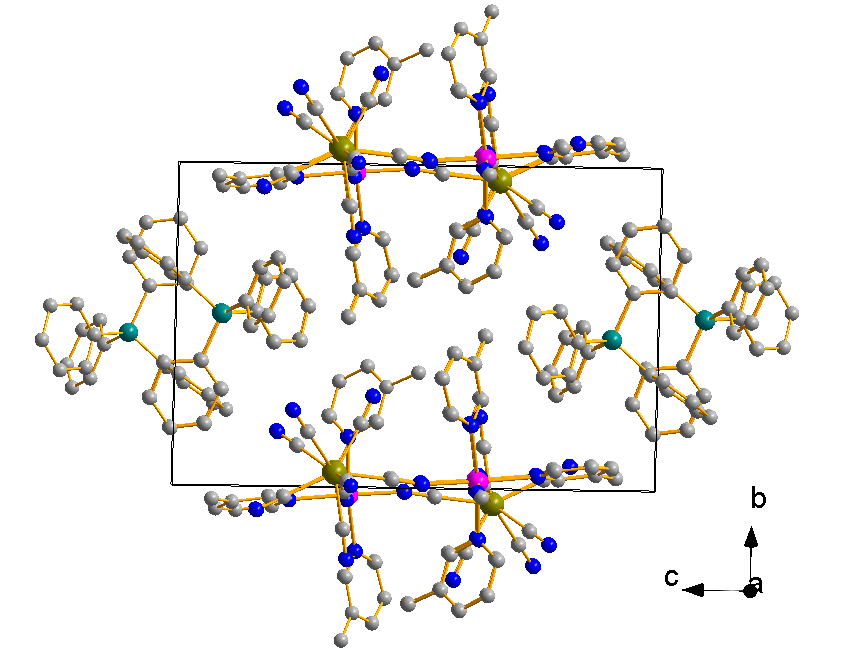


**Supplementary Figure S2.** The crystal packing of **1** and **2** viewed at the *a* axis. Color code: W^V^, dark yellow; Co^II^, pink; C, gray; N, blue; O, red; P or As, teal. Hydrogen atoms (**1** and **2**) and H_2_O molecules (**1)** were omitted for clarity.

**Supplementary Figure S3.** Variable-temperature dc magnetic susceptibility data in the form of *χ*_M_*T* for **2**, measured in an applied field of 2 kOe. The solid line in *χ*_M_*T* plot is the guide for eye. Inset: Plot of ln(*χ*_M_′*T*) *vs* *T*^-1^.

**Supplementary Figure S4.** Plots of zero-field cooled magnetization (ZFC) and field-cooled magnetization (FC) in a field of 10 Oe for **1**.

**Supplementary Figure S5.** Plots of zero-field cooled magnetization (ZFC) and field-cooled magnetization (FCM) in a field of 10 Oe for **2**.

**Supplementary Figure S6.** (a) Temperature dependence of in-phase (top) and out-of-phase (bottom) components of the ac susceptibility for **2** in zero applied static field with a 1Oe oscillating field at a frequency of 1-1500 Hz; (b) Cole-Cole diagram of **2**, plotted using *χ*_M_′ and *χ*_M_′′ at different temperature. The solid lines represent the fits to a general Debye model.

**Supplementary Figure S7.** Temperature dependence of in-phase (top) and out-of-phase (bottom) components of the ac susceptibility for **1** (a) and **2** (b) in zero applied static field with a 1 Oe oscillating field at a frequency of 1-1500 Hz (some of the plots were determined from 7 K to 11.8 K for saving time).

**Supplementary Figure S8.** (a) Magnetic hysteresis loops of polycrystalline **2** measured at 1.8, 2, 3, 5 and 10 K with a field sweep rate of 500 Oe/s. Solid line is guide for eyes; b) Hysteresis loops at 1.8 K on the oriented long crystal bundle of **2** along (black) and perpendicular (red) to the chain direction (*a* axis).


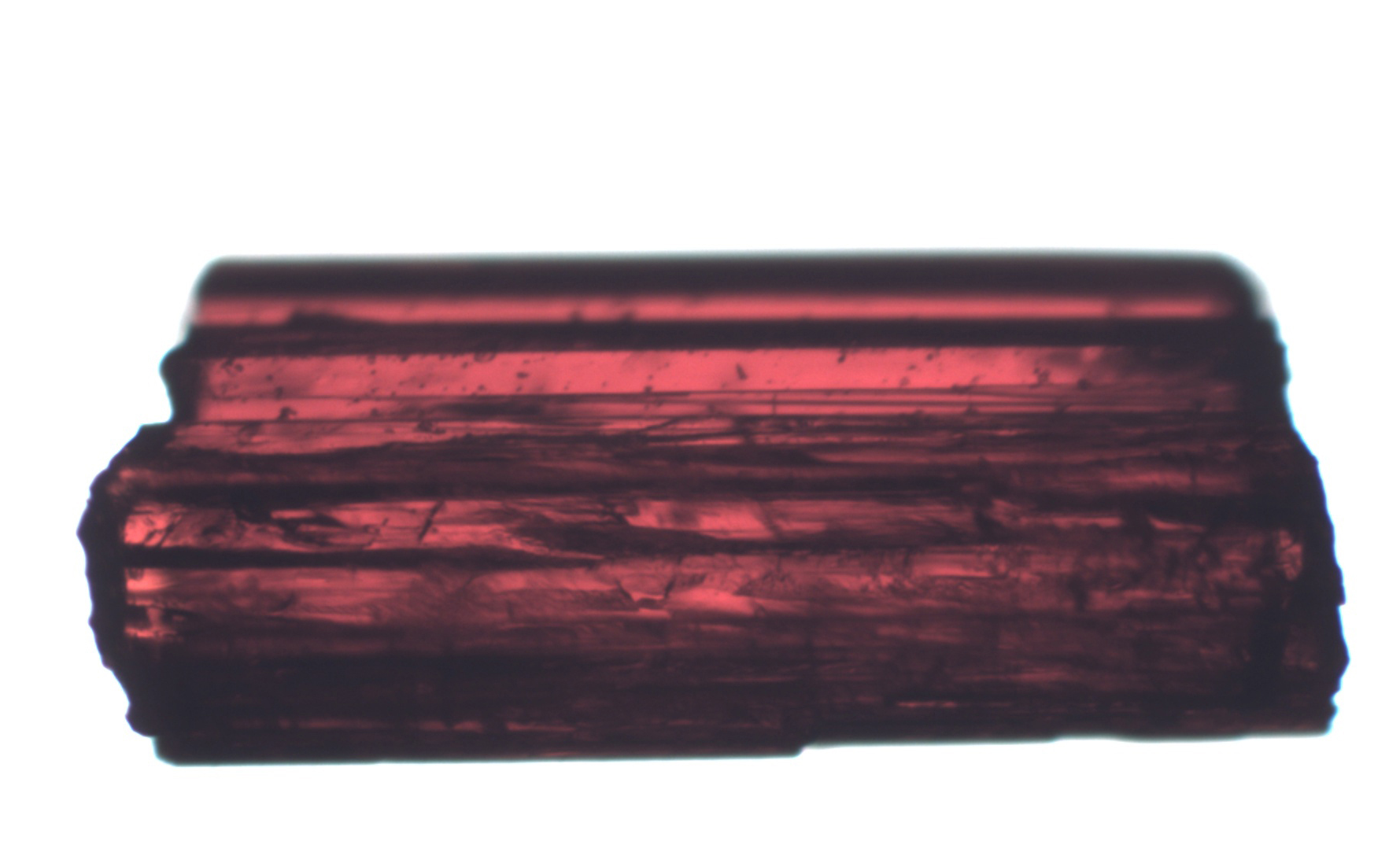


**Supplementary Figure S9.** Bundle of crystals suitable for anisotropic measurement of **1**.

**Supplementary Table S1. Crystallographic data for 1 and 2.**

|  | **1** | **2** |
| --- | --- | --- |
| empirical formula | C_48.2_H_40.7_N_10.7_O_0.9_CoWP | C_50_H_41_N_11_CoWAs |
| *M*_r_ | 1057.96 | 1113.64 |
| crystal system | Triclinic | Triclinic |
| space group | *P*$\mathop{1}\limits^{-}$ | *P*$\mathop{1}\limits^{-}$ |
| *a* /Å | 10.5166(6) | 10.560(4) |
| *b* /Å | 12.8081(7) | 13.036(4) |
| *c* /Å | 18.5932(10) | 18.803(6) |
| *α* / º | 84.308(2) | 84.554(5) |
| *β* / º | 74.162(2) | 74.188(5) |
| *γ* / º | 68.4840(10) | 68.166(5) |
| *V* /Å^3^ | 2241.5(2) | 2311.7(13) |
| *z* | 2 | 2 |
| *T /* K | 123(2) | 296(2) |
| *Ρ*_calc._/ mg m^-3^ | 1.568 | 1.600 |
| *λ* /Å | 0.71073 | 0.71073 |
| *μ* / mm^-1^ | 3.019 | 3.603 |
| *θ* / ° | 2.71-25.49 | 2.20-27.25 |
| crystal size / mm | 0.08 x 0.07 x 0.05 | 0.10 x 0.09 x 0.05 |
| F(000) | 1056 | 1104 |
| Index ranges | -12 ≤ *h* ≤ 9  -15 ≤ *k* ≤ 14  -22 ≤ *l* ≤ 22 | -11 ≤ *h* ≤ 12  -15 ≤ *k* ≤ 13  -22 ≤ *l* ≤ 22 |
| reflections collected | 18191 | 12286 |
| unique reflections | 8170 | 7933 |
| *R*_int_ | 0.0451 | 0.0325 |
| *R*_1_*^a^* [I>2*σ*(I)] | 0.0442 | 0.0712 |
| *ωR*_2_*^b^*(all data) | 0.1455 | 0.2177 |
| Largest diff. peak and hole (e.Å^-3^) | 1.439 and -1.001 | 1.992 and -3.496 |
| GOF on F^2^ | 1.099 | 1.038 |

*R*_1_*^a^* =*Σ*||*F*o|–|*F*c||/*Σ*|*F*o|；*wR*_2_*^b^*=[*Σ*[*w*(*F*o^2^ – *F*c^2^)^2^]/*Σ*[*w*(*F*o^2^)^2^]]^1/2^

**Supplementary Table S2.** Selected bond distances (Å), bond angles and W–C–N–Co torsion angles (°) for **1**.

| **1** | | | |
| --- | --- | --- | --- |
| W1–C1 | 2.164(8) | C1–N1 | 1.140(10) |
| W1–C2 | 2.162(8) | C2–N2 | 1.143(10) |
| W1–C3 | 2.181(8) | C3–N3 | 1.144(10) |
| W1–C4 | 2.178(9) | C4–N4 | 1.140(10) |
| W1–C5 | 2.196(10) | C5–N5 | 1.139(11) |
| W1–C6 | 2.189(10) | C6–N6 | 1.126(12) |
| W1–C7 | 2.160(8) | C7–N7 | 1.136(10) |
| W1–C8 | 2.142(10) | C8–N8 | 1.134(13) |
| Co1–O1 | 2.04(3) | Co1–N1#1 | 2.098(7) |
| Co1–N2 | 2.102(7) | Co1–N7#2 | 2.129(7) |
| Co1–N9 | 2.186(16) | Co1–N10 | 2.215(6) |
| Co1–N11 | 2.138(7) |  |  |
| O1–Co1–N1#1 | 96.8(8) | O1–Co1–N2 | 91.9(10) |
| N1#1–Co1–N2 | 90.6(3) | O1–Co1–N7#2 | 83.2(10) |
| N1#–Co1–N7#2 | 89.7(3) | N2–Co1–N7#2 | 175.1(3) |
| O1–Co1–N11 | 175.8(8) | N1#1–Co1–N11 | 86.7(3) |
| N2–Co1–N11 | 90.4(3) | N7#2–Co1–N11 | 94.5(3) |
| O1–Co1–N9 | 12.1(10) | N1#1–Co1–N9 | 85.9(4) |
| N2–Co1–N9 | 87.0(5) | N7#2–Co1–N9 | 88.2(5) |
| N11–Co1–N9 | 172.0(4) | O1–Co1–N10 | 84.9(8) |
| N1#1–Co1–N10 | 178.3(3) | N2–Co1–N10 | 89.2(2) |
| N7#2–Co1–N10 | 90.7(2) | N11–Co1–N10 | 91.6(3) |
| N9–Co1–N10 | 95.8(4) |  |  |
| C1–W1–C7 | 78.5(3) | C1–W1–C2 | 76.5(3) |
| C2–W1–C7 | 146.5(3) |  |  |
| Co1–N1#1–C1#1 | 170.2(7) | Co1–N2–C2 | 171.2(6) |
| Co1–N7#2–C7#2 | 159.2(7) |  |  |
| W1–C1–N1 | 178.6(7) | W1–C2–N2 | 175.1(7) |
| W1–C3–N3 | 178.5(8) | W1–C4–N4 | 178.3(8) |
| W1–C5–N5 | 178.3(8) | W1–C6–N6 | 177.8(10) |
| W1–C7–N7 | 176.0(7) | W1–C8–N8 | 177.1(10) |
| W1–C1–N1–Co1#1 | 163.756 | W1–C2–N2–Co1 | 170.293 |
| W1–C7–N7–Co1#3 | 152.405 |  |  |

Symmetry code: #1 = 2*-x*, *-y*, 1*-z*; #2 = *x*-1, *y*, *z*; #3=1+*x*, *y*, *z*.

***Supplementary Table S3.*** *Selected bond distances (Å), bond angles and W–C–N–Co torsion angles (°) for* ***2****.*

| Compound **2** | | | |
| --- | --- | --- | --- |
| W1–C1 | 2.170(17) | C1–N1 | 1.14(2) |
| W1–C2 | 2.090(13) | C2–N2 | 1.21(2) |
| W1–C3 | 2.164(19) | C3–N3 | 1.14(3) |
| W1–C4 | 2.15(2) | C4–N4 | 1.17(3) |
| W1–C5 | 2.165(17) | C5–N5 | 1.15(2) |
| W1–C6 | 2.18(2) | C6–N6 | 1.14(3) |
| W1–C7 | 2.182(17) | C7–N7 | 1.12(2) |
| W1–C8 | 2.16(3) | C8–N8 | 1.16(3) |
| Co1–N1 | 2.104(14) | Co1–N2#1 | 2.157(18) |
| Co1–N9 | 2.232(13) | Co1–N10 | 2.188(15) |
| Co1–N7#2 | 2.135(16) | Co1–N11 | 2.147(17) |
| N1–Co1–N7#2 | 90.0(6) | N1–Co1–N11 | 86.2(6) |
| N7#2–Co1–N11 | 91.3(7) | N1–Co1–N2#1 | 90.3(6) |
| N7#2–Co1–N2#1 | 174.0(6) | N11–Co1–N2#1 | 94.8(7) |
| N1–Co1–N10 | 86.9(7) | N7#2–Co1–N10 | 86.7(6) |
| N11–Co1–N10 | 172.8(7) | N2#1–Co1–N10 | 87.3(7) |
| N1–Co1–N9 | 178.3(6) | N7#2–Co1–N9 | 90.3(6) |
| N11–Co1–N9 | 92.1(6) | N2#1–Co1–N9 | 89.7(5) |
| N10–Co1–N9 | 94.7(7) |  |  |
| C2–W1–C1 | 79.1(6) | C1–W1–C7 | 76.8(7) |
| C2–W1–C7 | 145.5(6) |  |  |
| Co1–N1–C1 | 168.2(15) | Co1–N2#1–C2#1 | 159.9(14) |
| Co1–N7#2–C7#2 | 170.5(14) |  |  |
| W1–C1–N1 | 177.7(16) | W1–C2–N2 | 173.8(13) |
| W1–C3–N3 | 176(2) | W1–C4–N4 | 175.0(17) |
| W1–C5–N5 | 178(2) | W1–C6–N6 | 178.0(19) |
| W1–C7–N7 | 176.0(14) | W1–C8–N8 | 176(3) |
| W1–C1–N1–Co1 | 135.546 | W1–C2–N2–Co1#1 | 146.402 |
| W1–C7–N7–Co1#2 | 153.307 |  |  |

Symmetry code: #1=2-*x*, 2-*y*, 1-*z*; #2=1-*x*, 2-*y*, 1-*z*.

**Supplementary Table S4. Results of** **Continuous Shape Measure Analysis for [W^V^(CN)_8_]^3-^ units in 1 and 2*^a^***

| Geometry | SAPR-8 | BTPR-8 | TDD-8 | configuration |
| --- | --- | --- | --- | --- |
| **1** | 1.357 | 1.614 | 0.565 | TDD-8 |
| **2** | 1.304 | 1.303 | 0.662 | TDD-8 |

*^a^* The value are Continuous Shape Measure (CShM) parameters and CShM=0 for the ideal geometry and increases with the increase of the degree of distortion;

SAPR-8 corresponds to the square antiprism geometry;

BTPR-8 corresponds to the biaugmented trigonal prism geometry;

TDD-8 corresponds to the triangular dodecahedron geometry.

**Supplementary Table S5.** Fitted parameters of **1** for the Cole-Cole diagram using the generalized Debye model.

| *T* / K | *χ*_s_ (cm^3^mol^-1^) | *χ*_t_ (cm^3^mol^-1^) | *τ* / s | *α* | *R* |
| --- | --- | --- | --- | --- | --- |
| 9.2 | 4.09 | 94.48 | 0.06059 | 0.41 | 0.43 |
| 9.4 | 4.80 | 91.45 | 0.03836 | 0.40 | 0.53 |
| 9.6 | 5.72 | 88.57 | 0.02445 | 0.39 | 0.73 |
| 9.8 | 6.78 | 87.07 | 0.01644 | 0.39 | 1.57 |
| 10.0 | 8.24 | 85.45 | 0.01095 | 0.39 | 2.57 |
| 10.2 | 10.14 | 84.33 | 0.007383 | 0.38 | 3.60 |
| 10.4 | 12.94 | 83.14 | 0.004970 | 0.38 | 4.09 |
| 10.6 | 16.83 | 81.87 | 0.003273 | 0.37 | 4.58 |
| 10.8 | 22.06 | 80.54 | 0.002109 | 0.36 | 3.78 |
| 11.0 | 29.05 | 78.15 | 0.001306 | 0.35 | 2.94 |

**Supplementary Table S6.** Fitted parameters of **2** for the Cole-Cole diagram using the generalized Debye model.

| *T* / K | *χ*_s_ (cm^3^mol^-1^) | *χ*_t_ (cm^3^mol^-1^) | *τ* / s | *α* | *R* |
| --- | --- | --- | --- | --- | --- |
| 9.0 | 8.45 | 90.42 | 0.009972 | 0.40 | 3.57 |
| 9.2 | 9.45 | 91.88 | 0.007288 | 0.41 | 3.77 |
| 9.4 | 10.32 | 92.28 | 0.005311 | 0.41 | 4.14 |
| 9.6 | 11.33 | 91.94 | 0.003888 | 0.42 | 4.28 |
| 9.8 | 12.43 | 91.19 | 0.002823 | 0.42 | 4.52 |
| 10.0 | 13.92 | 90.16 | 0.002061 | 0.42 | 3.94 |
| 10.2 | 15.80 | 88.77 | 0.001497 | 0.42 | 3.83 |
| 10.4 | 18.21 | 87.00 | 0.001084 | 0.42 | 2.89 |
| 10.6 | 20.91 | 84.87 | 0.0007814 | 0.42 | 2.22 |
| 10.8 | 24.38 | 82.21 | 0.0005662 | 0.42 | 1.62 |
